# Electrodialysis with Bipolar Membranes for the Sustainable Production of Chemicals from Seawater Brines at Pilot Plant Scale

**DOI:** 10.1021/acssuschemeng.2c06636

**Published:** 2023-02-09

**Authors:** Calogero Cassaro, Giovanni Virruso, Andrea Culcasi, Andrea Cipollina, Alessandro Tamburini, Giorgio Micale

**Affiliations:** Dipartimento di ingegneria, Università degli studi di Palermo, Viale delle scienze Ed. 6, Palermo 90128, Italia

**Keywords:** BMED, brine mining, process intensification, electromembrane, ion-exchange membrane, circular economy, scale-up

## Abstract

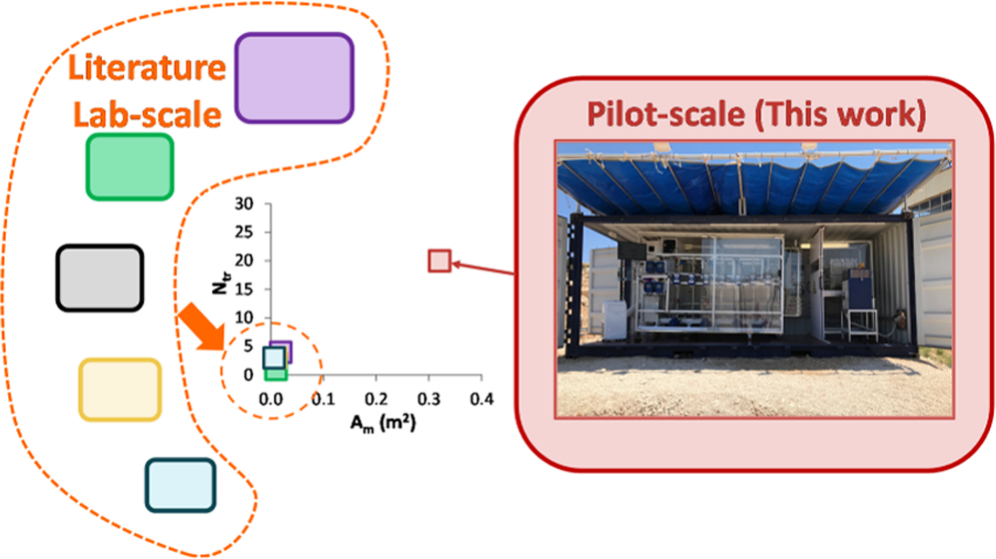

Environmental concerns regarding the disposal of seawater reverse osmosis brines require the development of new valorization strategies. Electrodialysis with bipolar membrane (EDBM) technology enables the production of acid and base from a salty waste stream. In this study, an EDBM pilot plant with a membrane area of 19.2 m^2^ was tested. This total membrane area results much larger (i.e., more than 16 times larger) than those reported in the literature so far for the production of HCl and NaOH aqueous solutions, starting from NaCl brines. The pilot unit was tested both in continuous and discontinuous operation modes, at different current densities (200–500 A m^–2^). Particularly, three different process configurations were evaluated, namely, closed-loop, feed and bleed, and fed-batch. At lower applied current density (200 A m^–2^), the closed-loop had a lower specific energy consumption (SEC) (1.4 kWh kg^–1^) and a higher current efficiency (CE) (80%). When the current density was increased (300–500 A m^–2^), the feed and bleed mode was more appropriate due to its low values of SEC (1.9–2.6 kWh kg^–1^) as well as high values of specific production (SP) (0.82–1.3 ton year^–1^ m^–2^) and current efficiency (63–67%). These results showed the effect of various process configurations on the performance of the EDBM, thereby guiding the selection of the most suitable process configuration when varying the operating conditions and representing a first important step toward the implementation of this technology at industrial scale.

## Abstract 2

Toward the sustainable production of hydrochloric acid and sodium hydroxide solutions from waste brines through the first electrodialysis with a bipolar membrane plant at pilot scale.

## Introduction

Freshwater scarcity is one of the greatest challenges that our generation must address. In recent years, the demand for freshwater has increased due to the growth of the global population^1^ and industrial activities.^2^ In response to this issue, seawater has been utilized as a raw material in producing freshwater.^3^ Global desalination capacity is approximately 95 million cubic meters per day.^4^ Thermal and membrane desalination processes are typically the world’s most widely used desalination technologies. Typical desalination technologies^5^ include multistage flash (MSF), multieffects distillation (MED), and reverse osmosis (RO) systems. Among these, reverse osmosis has been gaining increasing interest due to its lower consumption and reduced water cost compared to other desalination technologies thanks to efficient membranes, energy recovery systems, and suitable pretreatments.^6^ Thermal processes, such as MSF and MED, have a specific energy consumption (SEC) in the range of 6.5–25.5 kWh m^–3^ ^5^ and the cost of the produced freshwater is between $1.4^7^ and $1.1^8^ per m^3^ for MSF and MED, respectively. Reverse osmosis has a much lower consumption than thermal processes, ranging from 3 to 7 kWh m^–3^,^5^ and a cost of $0.75 per m^3^ of freshwater,^9^ which makes reverse osmosis the most adopted technology for seawater desalination.^10^ As a drawback, desalination technologies produce a concentrated stream known as waste brine.^11^ The waste brine is typically discharged into the sea,^12^ thus leading to possible environmental issues and relevant public concerns.^13^ In this regard, the scientific community has been devoting many efforts to proposing new technologies for valorizing waste brines generated by various industrial processes,^14^ desalination plants included. Ocean disposal,^15^ evaporation ponds,^16^ deep-well injection,^17,18^ and surface water discharge^19^ are the conventional methods for treating waste brines. However, using these technologies to treat brines has disadvantages associated with their high capital and negative environmental impact.^20^ Recently, membrane distillation (MD),^21,22^ membrane distillation crystallizers (MDCs),^23^ and reverse electrodialysis (RED)^24−26^ have been reported as innovative technologies to reduce the volume of waste brine, but are not able to recover value product from this waste, used individually. On the contrary, through minimum liquid discharge (MLD) or zero liquid discharge (ZLD) strategies, researchers have sought to minimize or eliminate brine disposal and valorize it.^27^ ZLD utilizes various technologies, including thermal processes, to eliminate wastewater streams.^28^ MLD systems generally integrate only membrane-based technologies, while ZLD systems implement both membrane-based and thermal-based technologies.^29^

Electrodialysis with bipolar membrane (EDBM)^30^ is an emerging electromembrane technology capable of producing acid and base solutions^31^ from the corresponding saline solution used as a feed.^32,33^ The repetitive unit (typically named triplet) of an EDBM system consists of a sequence of three channels hosting the electrolyte solutions separated by three ion-exchange membranes: anion (AEM), cation (CEM), and bipolar (BPM).^34,35^ Furthermore, two electrodes and an electrode rinse solution (ERS) are used to convert electricity into ionic current. When an electric potential is applied between the two electrodes, the water dissociation reaction takes place in the interlayer of the bipolar membrane, resulting in the production of protons and hydroxide ions.^36,37^ Due to the applied electric field, the ions are driven to opposite sides of the BPM based on their charge. Consequently, acidic and alkaline solutions are produced. A schematic representation of an EDBM unit (typically named stack) is provided in the supporting information (Figure S1).

Previous research focused on operating the EDBM under various operating conditions and process configurations. Reig et al.^12^ used synthetic NaCl solutions of various concentrations (100–200 g l^–1^) as the feed, operating the EDBM in a closed-loop configuration, applying a fixed voltage of 9 V. Concentrations of acid and base in the 0.66–2.2 M range were obtained, with current efficiency (CE) and SEC values ranging from 55 to 80% and 1.8 to 3.8 kWh kg^–1^ NaOH, respectively. Ibáñez et al.^38^ investigated the EDBM process using a synthetic RO concentrated solution that, apart from calcium and magnesium, mimicked the brine from a plant operating in Las Aguilas (Spain). The experiments were carried out in a closed-loop configuration with a fixed current density between 250 and 1000 A m^–2^. The acid and base produced had concentrations ranging from 0.6 to 1 M and current efficiencies ranging from 45 to 80%. Yang et al.^39^ fed an EDBM unit with a pretreated seawater reverse osmosis (SWRO) concentrate solution. In their work, the EDBM was operated at a constant current density of 570 A m^–2^ in a feed and bleed configuration at a flow rate of 0.3 L h^–1^ (for acid, base, and salt streams), producing a continuous stream of HCl and H_2_SO_4_ as acid and sodium hydroxide as base solutions. The reported concentration of protons produced was approximately 1 M, with CE and SEC values of 54% and 7.6 kWh kg^–1^, respectively. Hussain et al.^40^ proposed a two-stage batch process configuration operating at constant current, with the salt and acid solution being replaced with fresh feed and freshwater at the end of the first stage. In their configuration, the volume of the salt solution, made up of a synthetic NaCl stream, was 5 times that of the acid and base solution. With this configuration, they obtained an acid solution with concentrations ranging from 1.2 to 1.6 M and a concentrated base stream with a concentration of 3.4 M, with a relatively low SEC (i.e., 2.4–3.5 kWh kg^–1^) and a high CE (i.e., 42–60%), despite the high concentrations reached. Herrero-Gonzalez et al.^41^ also investigated the use of a synthetic NaCl solution to produce highly concentrated acid and base streams. Here, the EDBM was run in closed-loop operation mode for the acid and base compartments, while the salt compartment was operated in semibatch mode, with an intake of fresh solution and a purge. The obtained acid and base concentrations were up to 3.2 and 3.6 M, respectively, with higher values of SEC (i.e., 41 kWh kg^–1^ for acid) compared to the previous studies. Details on these works (e.g., configuration adopted, membrane area, and performance parameters) and those of the present work are summarized in the Supporting Information (Table S1 and Figure S2).

**Figure 1:**
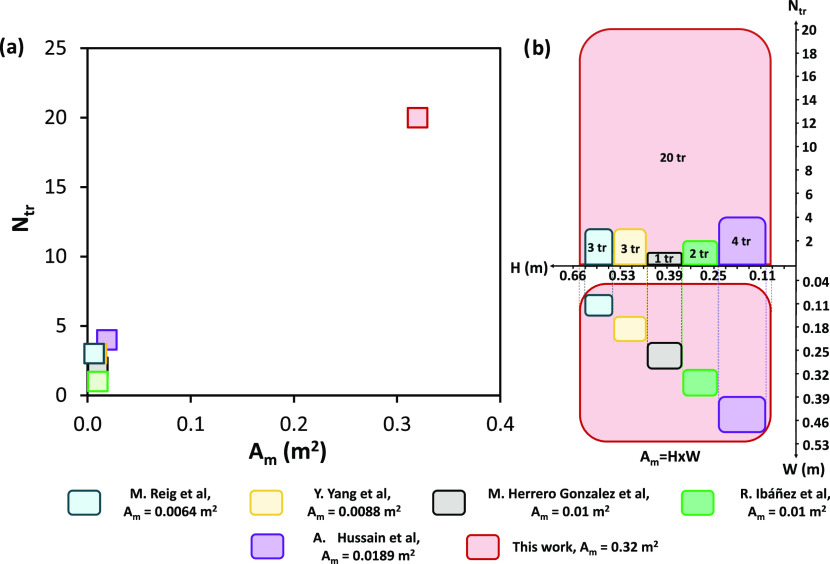
Comparison of the EDBM units investigated in previous literature with the unit presented in this work in terms of (a) number of triplets vs active membrane area and (b) scaled size representation. Red, this work; violet, Hussain et al.; green, Ibáñez et al.; gray, Herrero-Gonzalez et al.; yellow, Yang et al.; and cyan, Reig et al.

As shown in Figure 1, all of the previous studies were carried out on a laboratory scale with an active membrane area in the range of 0.0064–0.0189 m^2^ and a maximum total area of 0.23 m^2^.^40^ Additionally, the stacks were assembled with a few triplets, specifically between 1 and 4 (Figure 1a).

Moreover, although different process configurations (i.e., closed-loop, feed and bleed, fed-batch) were investigated individually, no previous works conducted a configuration comparison analysis, which is of particular interest for the industrial production of acid and base. The present work aims to present for the first time the behavior and performance of an up-scaled EDBM unit for producing HCl and NaOH solutions, starting from NaCl brines. In addition, a performance comparison of different process configurations (both in continuous and discontinuous modes of operation) is also presented for the first time at the pilot scale.

The EDBM pilot plant was realized as part of a larger treatment chain developed within the Horizon 2020 Water-Mining project.^42^ Some details on the project and on the treatment chain are provided in the Supporting Information.

## Description of the Pilot Plant Facility and Operational Procedures

In this section, the pilot plant installation site, the main element composing the EDBM pilot unit, and the operational and analytical procedures adopted during the tests are presented.

### Pilot Plant Installation Site

The EDBM unit was installed and tested on Lampedusa island (Italy), where the Water-Mining project demonstration treatment chain is located (see in Figure S3 in the Supporting Information). The demonstration system aims at treating the brine effluent generated by the local desalination plant (RO) to increase water recovery and promote mineral and chemical production using the ZLD strategy.

### Pilot Plant Description

This section presents the pilot plant assembling procedure, the EDBM equipment and membranes, the main instrumentations, the pumps, and acquisition and control systems.

### Pilot Plant Assembling

The pumping station frame was built with aluminum-profiled bars (METRA S.p.A.). In this work, 3/4 inch polypropylene (PP) pipes and fittings (FIP—Formatura Iniezione Polimeri S.p.A.) were used to build the hydraulic circuit that feeds the pilot plant. To retain coarse particles in suspension before being sent to the EDBM stack, a 3/4 inch polypropylene y-strainer filter (FIP—Formatura Iniezione Polimeri S.p.A.) and a cartridge filter with a 1 μm mesh (Atlas Filtri S.r.l.) were installed as pretreatment for the solutions. In the event of a potential liquid leak, a 6 cm high poly(vinyl chloride) basin was placed at the frame’s base. All of the materials were specifically chosen to ensure chemical and mechanical stability, as highly corrosive acid and base solutions are produced.

### EDBM Equipment and Membranes

The EDBM stack is an FT-ED1600-3 unit purchased from FuMA-Tech GmbH (Germany). In the EDBM stack, 40 triplets were installed, divided into two modules in a series, with 20 triplets each, reaching a total active membrane area of 19.2 m^2^. The unit is provided with the following ion-exchange membranes: FUMASEP FAB-PK anion-exchange membranes (poly(ethylene terephthalate) (PET) reinforced with polyketone (PK), 130 μm thick), FUMASEP FKB cation-exchange membranes (PK reinforced with PK, 130 μm thick), and FUMASEP FBM bipolar membranes (composite membrane reinforced with woven polyether ether ketone (PEEK), c. 160 μm thick). The recommended operational temperature range for the membranes is 15–40 °C. Each membrane has a membrane surface area of 0.454 × 0.345 m^2^. Additional information about the membrane properties is reported in Table S2. The spacers separating the membranes are woven type, made of PP, and 350 μm thick. The anode and cathode are dimensionally stable anode (DSA) and stainless steel, respectively.

### Instrumentation

Magnetic induction flowmeters (OPTIFLUX 4100C, KROHNE Messtechnik GmbH) were selected to measure stack inlet and outlet flow rates. The conductivities and pH values of the solutions are monitored using an inductive conductivity sensor (OPTISENS IND 1000, KROHNE Messtechnik GmbH) and a pH sensor (SMARTPAT PH 8320, KROHNE Messtechnik GmbH), respectively. Pressure transducers (OPTIBAR P 1010 C, KROHNE Messtechnik GmbH) were also installed at the inlet and outlet of the unit to evaluate the pressure drops along the stack.

### Pumps

Magnetically driven centrifugal pumps with a regenerative turbine (PTM 2.5 × 6, made in PP, TEOREMA S.r.l.) were selected to pump all of the solutions through the EDBM stack. These pumps were equipped with an external inverter to tune the flow rates. Two additional magnetically driven gear pumps (FG200–300 with 4 mm gears, made in AISI 316L, TEOREMA S.r.l.) were used for the acid and base line in feed and bleed and fed-batch configurations.

### Acquisition and Control Devices

The data acquisition hardware consists of a chassis (NI cDAQ-9179) and acquisition (C series: NI-9203, NI-9208) and command cards (C series: NI-9264, NI-9265, and NI-9266). LabVIEW software (National Instrument) is utilized for monitoring and controlling the pilot plant.

Continuous measurements of flow rate, conductivity, temperature, pressure, and pH were collected at the inlet and outlet of the EDBM stack for the three main solutions (i.e., acid, base, and salt). In contrast, the same measurements were collected only at the inlet for the electrode rinse solution (ERS). Details on the pumps and measurement devices are reported in the Supporting Information (Table S3).

### Plant Commissioning

**Figure 2:**
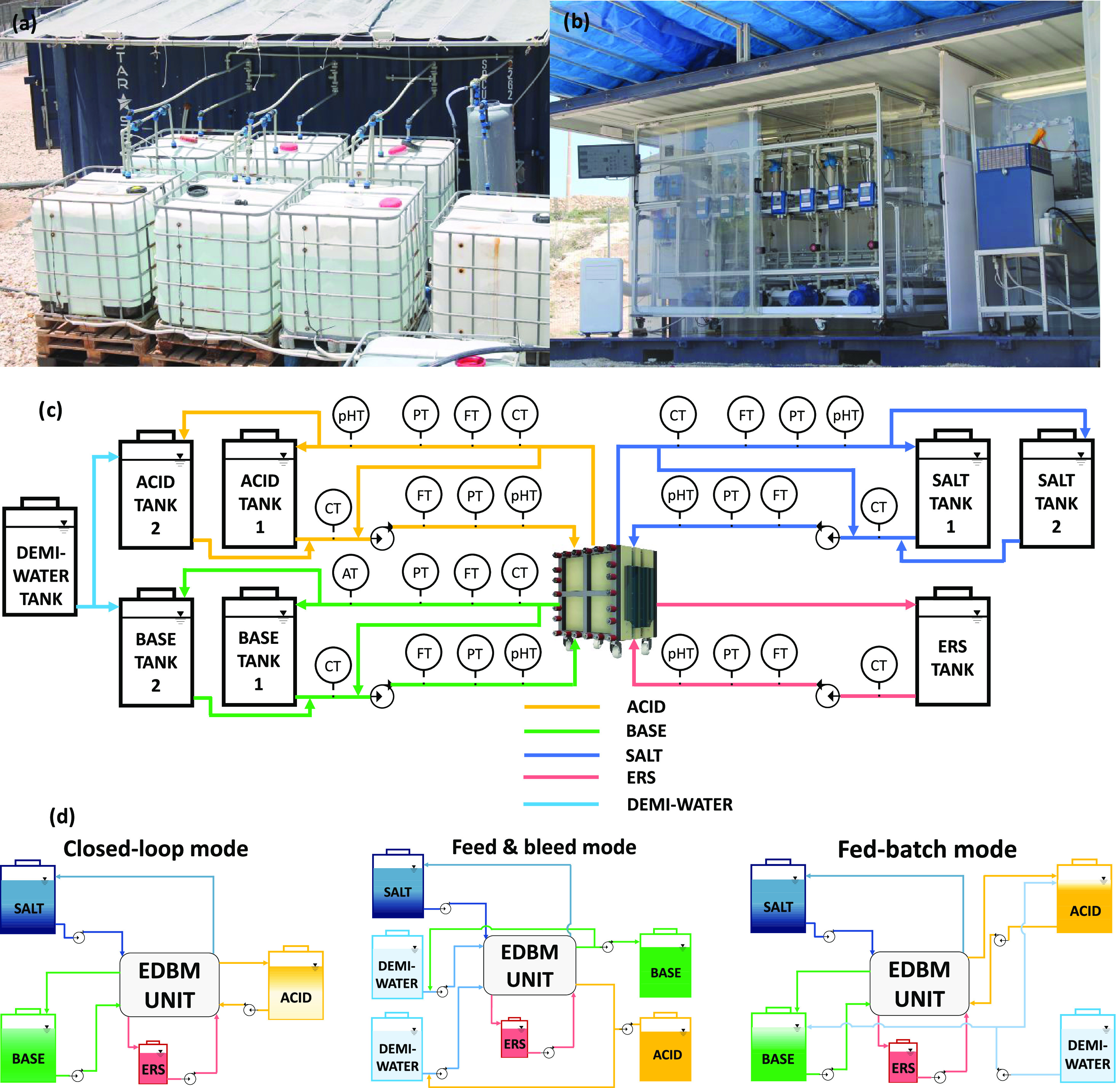
(a) Picture of the tank farm for storing the electrolyte solutions; (b) front view of the EDBM pilot unit and the relevant auxiliary components; (c) simplified process diagram of the EDBM pilot plant; and (d) schematics of the investigated process configurations: closed-loop (batch), feed and bleed (steady state),^43^ and fed-batch (batch).

The electrolytic solutions were stored in 1 m^3^ high-density polyethylene (HDPE) (IBC) tanks; conversely, the ERS was stored in a single 0.125 m^3^ polyethylene (PE) cylinder (Figure 2a). The stack was powered by a DC drive capable of delivering up to 17.5 kW (GIUSSANI S.r.l). An air conditioning unit and a fan were installed in the container (Figure 2b) to limit environmental temperature fluctuations. A simplified process flow diagram (PFD) of the EDBM pilot is depicted in Figure 2c. During the commissioning phase, external leakage tests on hydraulic circuits were performed up to the maximum expected pressure of 3 bar.

### Pilot Plant Configuration Schemes and Operational Procedures

The pilot plant was built to test three different process configurations: closed-loop, feed and bleed, and fed-batch modes (Figure 2d). In the closed-loop configuration, the solutions are pumped through the stack and recirculated until the desired product concentration is achieved (i.e., 1 M NaOH in this study). The salt solution volume was 3 times greater than the acid and base volumes to prevent the potential depletion of salt.

The feed and bleed configuration operates in continuous (steady-state) mode. The outlet stream from the stack is partially recirculated to the inlet, using an electrically actuated valve (VKDIV/CE DN15, made in PP, FIP—Formatura Iniezione Polimeri S.p.A.), to ensure sufficiently high channel flow velocities in the EDBM stack. The recirculation also allowed to reach the target concentration starting from demineralized water. Indeed, the mixing of the demi-water solution with the recirculated stream causes an increase in the conductivity of the EDBM inlet solutions. The outlet flow rates of acid and base depend on the utilized operating conditions (e.g., applied electric current) and can be tuned using the previously mentioned gear pumps.

The fed-batch configuration consists of two phases. First, the system is run in closed-loop mode for all compartments, with a small volume (i.e., 100 L) of acid and base. This procedure allows rapid increases in acid and base concentrations while maintaining high current efficiency and, thus, low specific energy consumption. Second, once the target base concentration was reached, the system was operated in a closed-loop configuration, with a makeup flow rate of water, in the acid and base tanks, in the range of 0.48–1.0 L min^–1^, depending on the applied current density. This makeup of water is provided to the tanks utilizing the two gear pumps mentioned above. In this way, during the second phase of operation, the acid and base concentrations were kept roughly constant while the volumes of the acid and base solutions were increasing. It is worth noting that the closed-loop and the fed-batch configurations are discontinuous, while the feed and bleed is a continuous mode of operation.

The reference flow rate through the stack for the acid, base, and salt solutions was 5 L min^–1^, and for the ERS was 20 L min^–1^. These flow rates correspond to a mean channel flow velocity of 2.6 cm s^–1^. The stack was cleaned after each experiment by pumping demineralized water for 20 min, using a flow rate of 2 L min^–1^. EDBMs can be damaged if the concentration of Ca^2+^ and Mg^2+^ ions in the streams exceeds 10 ppm. As a result, the demineralized water used to clean the stack contains less than 3 ppm of Ca^2+^ and Mg^2+^. The cleaning time of 20 min was chosen to achieve at the stack outlet conductivities low enough (350 μS cm^–1^) to guarantee the absence of acid and base. In addition, an acid and base cleaning is performed once a month to prevent scaling issues, using hydrochloric acid and sodium hydroxide solutions (5 wt %). Each test was carried out at room temperature (c. 25 °C), and temperature effects were observed. Nonetheless, the temperature of the solutions was always lower than the limits suggested by the supplier (15–40 °C) during all tests.

### Solution Preparation, Sampling, and Analytical Procedures

The starting solutions were prepared with high-quality chemicals and distilled water. For each test, 1 m^3^ saline solution with a concentration of 1 M NaCl (>99.5% purity, Saline di Volterra S.r.l) was prepared (with impurities of Ca^2+^ and Mg^2+^ < 10 ppm). This concentration and the presence of NaCl only are chosen in accordance with the features of the brine coming upstream the EDBM unit in the treatment chain of the water-mining project (see the Supporting Information and Figure S4). A volume of 0.3 and 0.1 m^3^ of acid and base solutions were prepared for each closed-loop and fed-batch tests, respectively, with 0.05 M of HCl (ACS reagent 37 wt %, Honeywell, Fluka) and NaOH (technical grade, Inovyn). This initial concentration of 0.05 M was employed to increase the conductivities of acid and base solutions preventing the application of high voltage to the stack at the beginning of the tests. Furthermore, an ERS of 0.125 m^3^ with a concentration of 0.25 M of Na_2_SO_4_ (technical grade, CR GRUPO CRIMIDESA) solution was used. The ERS was replaced after each test: this was done because the ERS pH was observed to decrease during the tests due to the high mobility of protons (see Figure S5 in the Supporting Information). During testing, acid and base samples of 50 mL were collected once per hour for the analytical characterization. Titration analyses were performed for the acid and base solutions using Na_2_CO_3_ (0.05 M) and HCl (0.1 M), respectively, using methyl orange as an indicator. Regarding the salt solution, only the conductivity was measured.

### Performance Indicators

Different performance indicators were employed to analyze the performance of the EDBM unit operated with the three configurations.

Yield (τ_p_) represents the ratio of the produced quantity of NaOH or HCl to the initial or inlet amount of NaCl. Equations 1 and 2 are used for a discontinuous and a continuous mode of operation respectively,
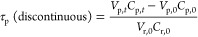
1
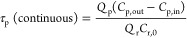
2where the subscripts *t* and 0 mean at time *t* and at the beginning of the test, respectively, the subscripts in and out refer to the inlet and outlet, respectively, and the subscripts p and r indicate the product (i.e., acid or base) and the reagent (i.e., salt), respectively. *V*_p,*t*_, *V*_p,0_, and *V*_r,0_ are the corresponding volumes of the solutions (m^3^); *C*_p,*t*_, *C*_p,0_, *C*_p,out_, *C*_p,in_, and *C*_r,0_ are the concentrations (mol L^–1^); *Q*_p_ and *Q*_r_ are the outlet flow rate of the product solution and the inlet flow rate of the reagent (L min^–1^), respectively.

Current efficiency (CE, %) accounts for the amount of electric charges introduced into the system which was successfully converted for the production of protons or hydroxide ions. Equations 3 and 4 are used for a discontinuous and a continuous mode of operation, respectively,
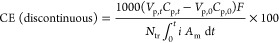
3
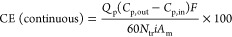
4where *F* is Faraday’s constant (i.e., 96 485 C mol^–1^), *N*_tr_ is the triplet number, *A*_m_ is the membrane active area (m^2^), and *i* (A m^–2^) is the electric current density provided to the stack.

Specific energy consumption (SEC, kWh kg^–1^) is the energy consumed to produce 1 kg of the desired product (i.e., NaOH or HCl). Equations 5 and 6 are used for a discontinuous and a continuous mode of operation, respectively,
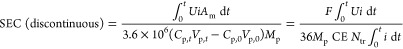
5
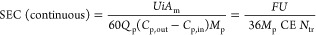
6where *U* is the electric potential (V) applied to the stack and *M*_p_ is the molar mass of the desired product (g mol^–1^).

Specific production (SP, ton year^–1^ m^–2^) indicates the mass of product (i.e., NaOH or HCl) produced in a working year (8000 working hours are assumed in this study) per unit of the total membrane area. Equations 7 and 8 are used for a discontinuous and a continuous mode of operation, respectively,
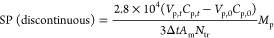
7
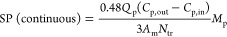
8where Δ*t* is the process time (s).

## Results and Discussion

Each of the three process configurations was tested by operating the EDBM pilot at two different current densities (i.e., either 200 or 400 A m^–2^). The three operation mode results will be discussed separately and compared in the last section (Table 1).

**Table 1:** Summary of the Tests Performed in This Work with the EDBM Pilot Plant in Terms of Process Configurations, Current Density, and Initial Conditions of the Four Solutions^a^

test	configuration	*i* (A m^–2^)	acid line	base line	salt line	ERS line
1	closed-loop	200	*V*_p,0_ = 0.3	*V*_p,0_ = 0.3	*V*_r,0_ = 0.9	*V*_ERS_ = 0.125
			*C*_p,0_ = 0.05	*C*_p,0_ = 0.05	*C*_r,0_ = 1	*C*_ERS_ = 0.25
			*Q* = 5	*Q* = 5	*Q* = 5	*Q* = 20
2	closed-loop	400	*V*_p,0_ = 0.3	*V*_p,0_ = 0.3	*V*_r,0_ = 0.9	*V*_ERS_ = 0.125
			*C*_p,0_ = 0.05	*C*_p,0_ = 0.05	*C*_r,0_ = 1	*C*_ERS_ = 0.25
			*Q* = 5	*Q* = 5	*Q* = 5	*Q* = 20
3	feed and bleed	200	*C*_p,in_ = 0	*C*_p,in_ = 0	*V*_r,0_ = 0.9	*V*_ERS_ = 0.125
			*Q*_p_ = 0.48	*Q*_p_ = 0.48	*C*_r,0_ = 1	*C*_ERS_ = 0.25
			*Q*_rec_ = 4.52	*Q*_rec_ = 4.52	*Q* = 5	*Q* = 20
4	feed and bleed	400	*C*_p,in_ = 0	*C*_p,in_ = 0	*V*_r,0_ = 0.9	*V*_ERS_ = 0.125
			*Q*_p_ = 1	*Q*_p_ = 1	*C*_r,0_ = 1	*C*_ERS_ = 0.25
			*Q*_rec_ = 4	*Q*_rec_ = 4	*Q* = 5	*Q* = 20
5	fed-batch	200	*V*_p,0_ = 0.1	*V*_p,0_ = 0.1	*V*_r,0_ = 0.9	*V*_ERS_ = 0.125
			*C*_p,0_ = 0.05	*C*_p,0_ = 0.05	*C*_r,0_ = 1	*C*_ERS_ = 0.25
			*Q* = 5	*Q* = 5	*Q* = 5	*Q* = 20
			*Q*_water_ = 0.5	*Q*_water_ = 0.5		
6	fed-batch	400	*V*_p,0_ = 0.1	*V*_p,0_ = 0.1	*V*_r,0_ = 0.9	*V*_ERS_ = 0.125
			*C*_p,0_ = 0.05	*C*_p,0_ = 0.05	*C*_r,0_ = 1	*C*_ERS_ = 0.25
			*Q* = 5	*Q* = 5	*Q* = 5	*Q* = 20
			*Q*_water_ = 1	*Q*_water_ = 1		
7	closed-loop	300	*V*_p,0_ = 0.3	*V*_p,0_ = 0.3	*V*_r,0_ = 0.9	*V*_ERS_ = 0.125
			*C*_p,0_ = 0.05	*C*_p,0_ = 0.05	*C*_r,0_ = 1	*C*_ERS_ = 0.25
			*Q* = 5	*Q* = 5	*Q* = 5	*Q* = 20
8	closed-loop	500	*V*_p,0_ = 0.3	*V*_p,0_ = 0.3	*V*_r,0_ = 0.9	*V*_ERS_ = 0.125
			*C*_p,0_ = 0.05	*C*_p,0_ = 0.05	*C*_r,0_ = 1	*C*_ERS_ = 0.25
			*Q* = 5	*Q* = 5	*Q* = 5	*Q* = 20
9	feed and bleed	300	*C*_p,in_ = 0	*C*_p,in_ = 0	*V*_r,0_ = 0.9	*V*_ERS_ = 0.125
			*Q*_p_ = 0.75	*Q*_p_ = 0.75	*C*_r,0_ = 1	*C*_ERS_ = 0.25
			*Q*_rec_ = 4.25	*Q*_rec_ = 4.25	*Q* = 5	*Q* = 20
10	feed and bleed	500	*C*_p,in_ = 0	*C*_p,in_ = 0	*V*_r,0_ = 0.9	*V*_ERS_ = 0.125
			*Q*_p_ = 1.2	*Q*_p_ = 1.2	*C*_r,0_ = 1	*C*_ERS_ = 0.25
			*Q*_rec_ = 3.8	*Q*_rec_ = 3.8	*Q* = 5	*Q* = 20

a *V* (m^3^), *C* (mol L^–1^), and *Q* (L min^–1^).

### Closed-Loop Configuration

**Figure 3:**
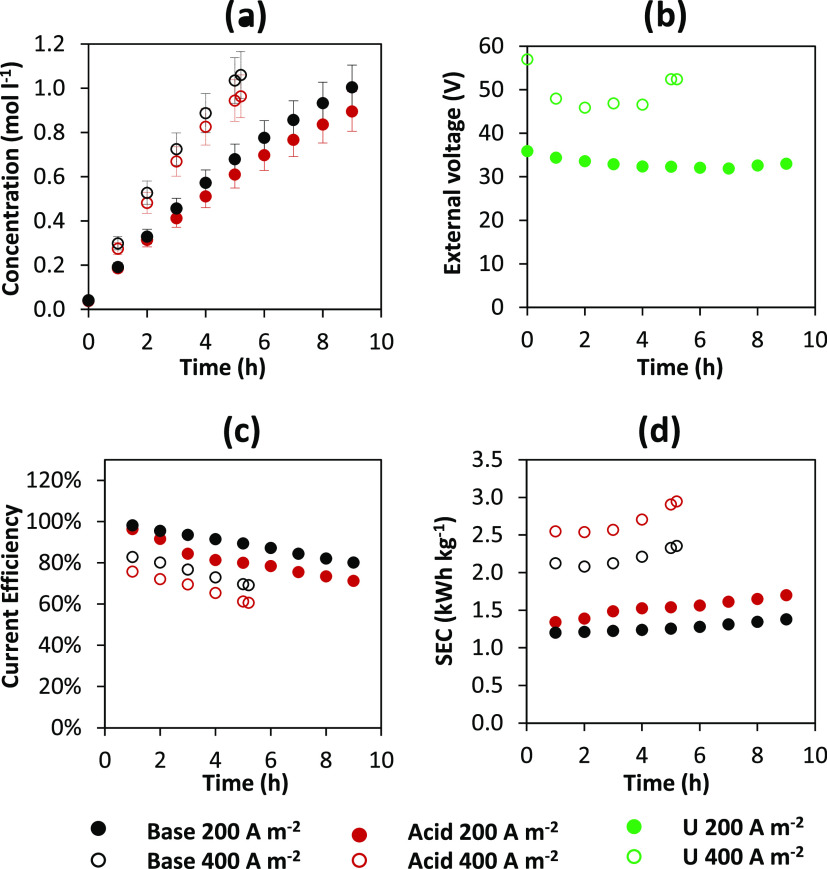
Time-dependent profiles of (a) HCl and NaOH concentrations, (b) external voltage, (c) current efficiency, and (d) specific energy consumption for acid and base for tests performed at 200 and 400 A m^–2^. Pilot operation mode: closed-loop (batch).

Figure 3 shows the acid/base concentrations, voltage, CE, and SEC as functions of time when the applied current density was either 200 or 400 A m^–2^.

As shown in Figure 3a, the acid and base concentrations vary linearly with time only during the first hours of the test (about 4 h). Then, a less-than-linear trend, on the other hand, is observed as a result of a decrease in acid and base production over time due to nonideal membrane permselectivity, osmosis, electro-osmosis and parasitic current phenomena.^44^ This graph shows that the base concentration increases faster than the acid concentration for both the electric currents investigated. This phenomenon is most likely due to the different effects of the acid and base diffusion phenomena on the saline solution channel. As a result of the high mobility of the protons, acid diffusion is presumably dominant. The pH in the salt channel falls from around 9 at the beginning of the tests to less than 2 toward the end, and the pH of ERS reduces from 2.3 to 1.1 (Figure S5). Due to these nonideal phenomena, a volume variation of approximately 17% was observed in the acid and base tank. The final concentrations at 200 A m^–2^ are 0.895 and 1 M for the acid and base solutions, respectively, and 0.964 and 1.06 M for the acid and base solutions at 400 A m^–2^ (Figure 3a). As a result, the target concentration (i.e., 1 M NaOH) was met in both cases. Specifically, the target was attained in 9 h at 200 A m^–2^ and 5.2 h at 400 A m^–2^. The process time to achieve the 1 M NaOH target at 400 A m^–2^ is expected to be half of that at 200 A m^–2^. Conversely, in the 400 A m^–2^ test, the process time is approximately 0.56 times that of the test conducted at 200 A m^–2^. This fact is not surprising, given that higher current density reduces current efficiency (Figure 3c). Since the volume of the salt is 3 times that of the acid and base, the yield value at 1 M concentration of product should be around 33%. At the same target concentration of NaOH, however, an increase in the volume of the alkaline solution due to electro-osmosis effects is observed during the test, resulting in a yield greater than 38%. The volume of the acid solution also increases during the test, but the greater weight of the diffusive phenomena results in a lower yield. The current efficiency of the base and the acid shows a decreasing and monotonous trend (Figure 3c). At 200 A m^–2^, the current efficiency is in the range of 80–100% for the base and 96–71% for the acid, while at 400 A m^–2^ is in the range of 83–69% for the base and 76–61% for the acid. Nonideal phenomena such as diffusion, electro-osmosis, and parasitic currents via manifolds cause a reduction in current efficiency over time in closed-loop tests. Regardless of the applied current density, the average difference between acid and base is about 0.1 mol L^–1^ due to the greater extent of acid diffusion than the base diffusion. The interpretation of the SEC results (Figure 3d) is directly related to the current efficiency and electric potential values (according to eq 5). Regardless of the applied electric current, the SEC trend is increasing over time. The SEC profile, in particular, is practically linear in the test at 200 A m^–2^, whereas it is nonlinear at 400 A m^–2^, with a sudden increase in slope after about 3 h of operation. Indeed, the effect of two opposing phenomena occurs: on the one hand, a decrease in potential over time, and on the other hand, a reduction in current efficiency. The latter phenomenon prevails, causing the SEC to rise over time. At the end of the test, the SEC values are 1.7 and 1.4 kWh kg^–1^ for acid and base, respectively, at 200 A m^–2^ and 2.94 and 2.35 kWh kg^–1^ for acid and base, respectively, at 400 A m^–2^. The voltage shows an overall decreasing trend over time due to two opposing phenomena: increasing the acid and base concentrations during the test causes a decrease in the average electrical resistance of the stack while increasing the Nernst potential. Overall, the reduction in the stack’s electric resistance dominates, determining the downward trend of the electric potential. At 200 A m^–2^, the average electric potential is 33.1 V, and at 400 A m^–2^, it is 49.9 V. The voltage increase at the end of the test at 400 A m^–2^ may be due to the higher acid and base concentrations reached, which caused a greater depletion in the salt channel. Indeed, the lower salt concentration caused a higher stack resistance and thus the increase in stack voltage. Finally, the average specific production for the test at 200 A m^–2^ is 0.63 ton year^–1^ m^–2^ for the base and 0.51 ton year^–1^ m^–2^ for the acid. At 400 A m^–2^, the base has an average productivity of 1.1 ton year^–1^ m^–2^ and the acid has an average productivity of 0.88 ton year^–1^ m^–2^. The SP is less than double at 400 A m^–2^ compared to that at 200 A m^–2^, as a result of the lower current efficiency for both acid and base.

### Feed and Bleed Configuration

**Figure 4:**
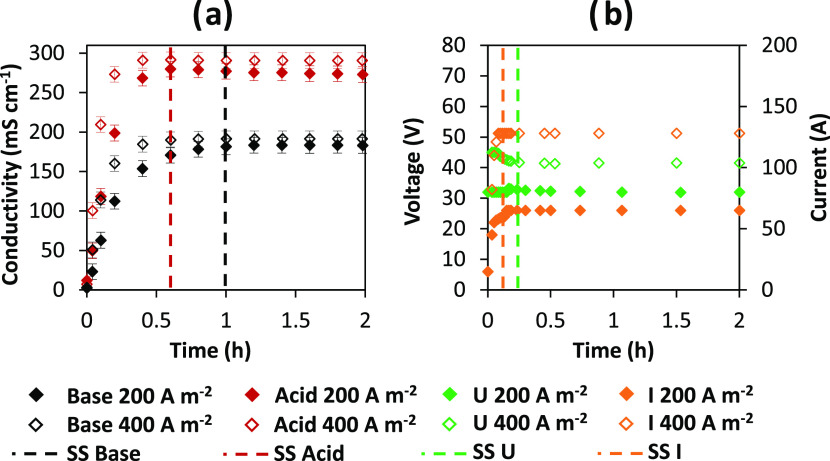
Time-dependent profiles of (a) acid and base electric conductivities and (b) external voltage and current for tests performed at 200 and 400 A m^–2^. Vertical dashed lines indicate the end of the transient startup of the pilot. Pilot configuration: feed and bleed.

This section reports the results relevant to the continuous steady-state operation of the EDBM pilot at the two investigated current densities. In this case, the inlet acid and base solutions are made up of demineralized water. The target concentration of NaOH can be achieved in this case by selecting appropriate operating conditions in terms of flow rate (outlet and recirculated streams) and electric current. Figure 4 depicts the time dependency of the solution conductivity on the left and the electric current and voltage on the right.

At the beginning of these tests, a potentiostatic mode is used to avoid excessive voltage rise due to the low conductivity of the inlet demineralized water streams. After about 0.15 h, the electric current stabilizes, and the operating mode is switched from potentiostatic to galvanostatic. The voltage remains nearly stable after this transient startup, with a 1 V drop throughout the test. At both applied current densities, the desired NaOH concentration is achieved using this configuration by suitably tuning the outlet and recirculated stream flow rates. The main performance parameters and outlet flow rates for the two current densities are summarized in Table 2.

**Table 2:** Summary of the Results Obtained in the Feed and Bleed Configuration at 200 and 400 A m^–2^

*i* (A m^–2^)	*Q*_bleed_ (L min^–1^)	CE (%)	SEC (kWh kg^–1^)	SP (ton year^–1^ m^–2^)	*C* (mol L^–1^)
200	0.48	59.7	1.8	0.48	1.01
400	1	66.3	2.1	1.06	1.06

Remarkably, in contrast to the closed-loop configuration, the SEC at 400 A m^–2^ is only 17% higher than at 200 A m^–2^. Furthermore, at 400 A m^–2^, the current efficiency is 11% higher (in absolute values) than at 200 A m^–2^. Interestingly, this results in more than double specific production at 400 A m^–2^ than that at 200 A m^–2^.

### Fed-Batch Configuration

**Figure 5:**
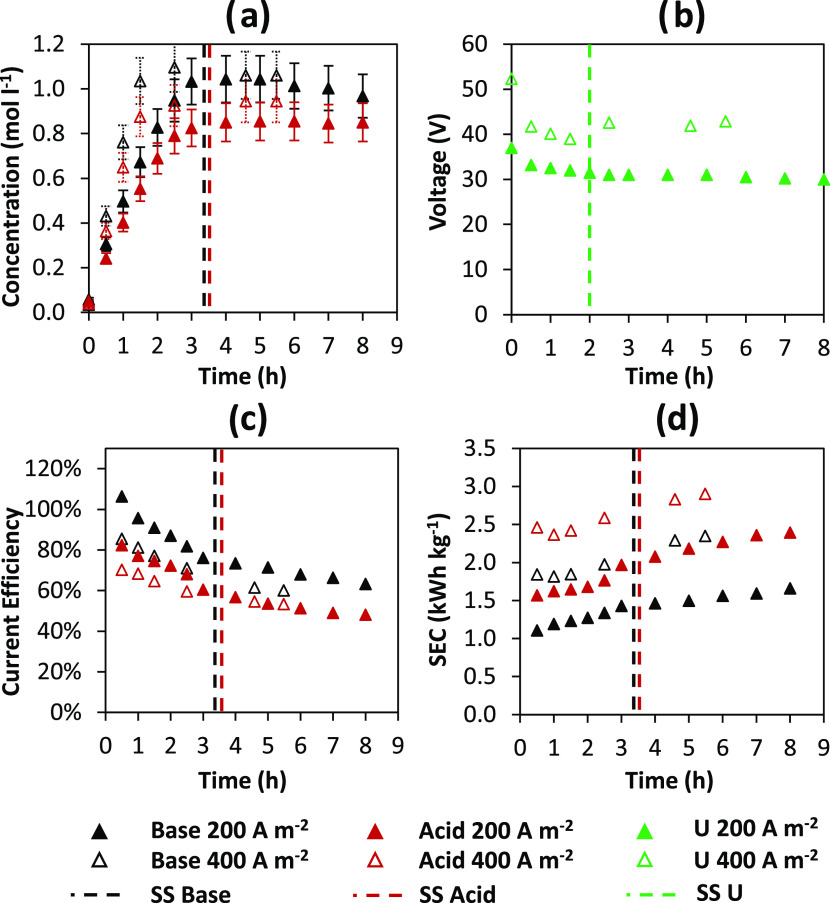
Time-dependent profiles of (a) HCl and NaOH concentrations, (b) external voltage, (c) current efficiency, and (d) specific energy consumption of acid and base for tests performed at 200 and 400 A m^–2^. Vertical dashed lines indicate the end of the closed-loop phase and the startup of the fed-batch phase. Pilot operation mode: fed-batch (batch).

Figure 5 shows the concentration profiles, current efficiency, and SEC for acid and base, as well as the voltage applied to the stack as functions of time.

Since the system is operated in a closed-loop configuration, the acid and base concentrations increase in the first part of the test and remain nearly constant in the second part. Indeed, in the final part of the test, the system operates similarly to the batch configuration (i.e., the conventional closed-loop configuration) but with a demi-water inlet makeup stream feeding the acid and base tanks. In both tests, the final concentration of base is slightly higher than 1 M, precisely 1.04 and 1.06 M for tests performed at 200 and 400 A m^–2^, respectively (Figure 5a). On the contrary, the final acid concentration reached was 0.85 and 0.95 M, respectively (Figure 5a). Likewise to the results obtained in the closed-loop configuration (see Closed-Loop Configuration section), the acid concentration is lower than the base concentration due to larger diffusion fluxes across the AEM than the CEM, which ultimately causes a decrease in the current efficiency for the acid (Figure 5c). Furthermore, the higher acid concentration at 400 A m^–2^ is due to the higher applied current density. Regardless of the current density used, the makeup stream of water is sent to the acid and base tanks around the third hours of operation. Because of the small volume of solution used, concentrations rise rapidly in the first three hours of operation, as shown in Figure 5a. As a result of the diluting effect caused by the addition of makeup water, the concentration profiles for both the acid and base flatten. The water makeup flow rate was chosen to ensure that the concentration remained quasi-stationary. However, a slightly decreasing trend in concentration is observed, particularly for the base, while the acid concentration is approximately stable. At both 200 and 400 A m^–2^, the electrical potential is somehow more stable than that obtained with the conventional closed-loop configuration (see Figure 3), especially once the water makeup enters the acid and base tanks. Since the initial conditions are the same, the starting voltage values (Figure 5b) are similar to those obtained in the closed-loop configuration. However, the average value of the electric potential is lower because the system operates at a higher concentration for most of the test time than the conventional closed-loop configuration. Indeed, regardless of the applied current density, the system operates in quasi-steady-state conditions with average concentrations of 0.89 and 1 M for acid and base. The advantage of having a lower average applied voltage is offset by a decrease in current efficiency due to the increased weight of nonideal phenomena such as diffusion, water transport, and parasitic currents. These phenomena are exacerbated when the system operates at high concentrations and/or concentration gradients across the membranes. At the end of the test, the current efficiency was 63.2 and 60% at 200 and 400 A m^–2^ for the base, respectively, and 48 and 53% at 200 and 400 A m^–2^ for the acid, respectively, (Figure 5c). Similar to the conventional closed-loop case, the lower concentration of acid implies a lower current efficiency than the base. In particular, in comparison to the base, the acid has an average reduction of 22% (in absolute values) of current efficiency and an average value of 50.5%. The SEC reflects the current efficiency and voltage values (Figure 5d). Regardless of the applied current, the final SEC values were 1.7–2.4 kWh kg^–1^ for the base and 2.4–2.9 kWh kg^–1^ for the acid. Despite the decrease in average applied potential, the reduction in observed current efficiency determines the increase in SEC values compared to the conventional closed-loop case. Finally, the obtained SPs for the base are 0.5 and 0.96 ton year^–1^ m^–2^ at 200 and 400 A m^–2^, respectively, and 0.35 and 0.78 ton year^–1^ m^–2^ for the acid at 200 and 400 A m^–2^, respectively.

### Comparison between the Three Process Configurations

**Figure 6:**
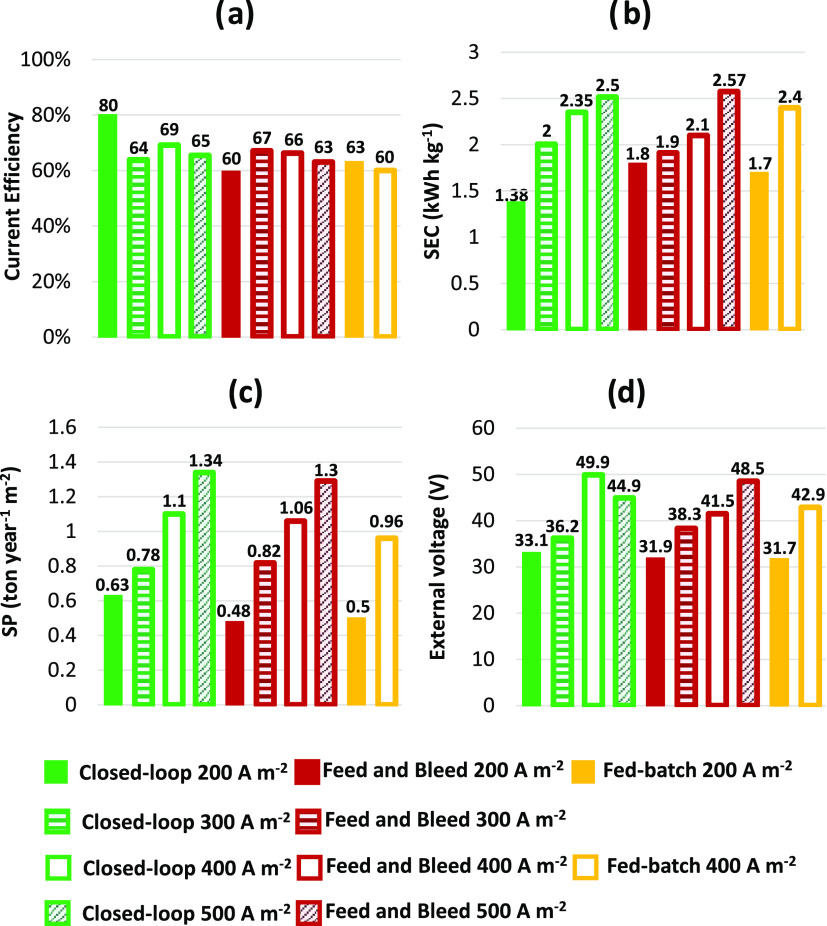
Histogram charts of the NaOH-based (a) current efficiency (CE), (b) specific energy consumption (SEC), (c) specific production (SP), and (d) external voltage for the closed-loop and feed and bleed configurations at 200, 300, 400, and 500 A m^–2^ as well as for the fed-batch configuration at 200 and 400 A m^–2^, calculated at 1 M NaOH target concentration.

This paragraph presents a comprehensive comparison analysis of the performance parameters for three investigated configurations (Figure 6). This analysis focuses on the target product only (i.e., sodium hydroxide), while ignoring the secondary product, hydrochloric acid.

The closed-loop configuration performed better in terms of CE, SEC, and SP at 200 A m^–2^. In particular, a CE of 25 and 21% higher was discovered in comparison to the two other investigated process configurations (i.e., feed and bleed and fed-batch). In contrast to the feed and bleed and fed-batch configurations, the closed-loop configuration operates for the majority of the process time with average concentrations that are lower than the target. Consequently, there is an increase in current efficiency as a result of the attenuation of nonideal phenomena, which are exacerbated at higher concentrations. In comparison to feed and bleed and fed-batch configurations, the increased current efficiency in the closed-loop configuration at 200 A m^–2^ reduces specific consumption by 30 and 23%, respectively. In addition, the higher average voltage (Figure 3d) obtained in the closed-loop configuration has a smaller effect on the SEC than the effect on CE. When operating in the closed-loop configuration, the achieved SPs are 24 and 21% higher than the other two configurations. As a result of this analysis, the closed-loop configuration is preferred at 200 A m^–2^. When the electric current density is increased to 400 A m^–2^, there are no significant differences in current efficiency values among the three process configurations studied (less than 13% of relative variation). Specific production at 400 A m^–2^ is 3.6 and 10% higher in the closed-loop configuration than in the feed and bleed and fed-batch configurations, respectively. However, the SEC values in the feed and bleed configuration are 13% lower than in the other two configurations. Overall, due to the favorable performance parameters and the fact that it operates in steady-state conditions, the feed and bleed configuration would be preferred at 400 A m^–2^. Indeed, steady-state conditions ensure more stable production and safer working conditions than discontinuous configurations. According to the reported results, the fed-batch configuration is preferred in neither of the investigated current densities. At 400 A m^–2^, there is no discernible difference in performance parameters between closed-loop and feed and bleed. As a result, more tests were conducted to investigate different values of current densities around 400 A m^–2^. Four additional tests (tests 7–10, Table 1) were carried out, specifically at 300 and 500 A m^–2^ (whose results are reported in the Supporting Information for brevity: Figure S6 and Table S4) using the closed-loop and feed and bleed configurations. The additional tests confirm that there are no significant differences in the qualitative trends of the results (Figure S7). The results showed that closed-loop is the best performing configuration at low currents, while feed and bleed shows comparable or even better performance at higher current densities. Overall, the data collected indicate that the feed and bleed configuration might be slightly preferable.

Except for the initial startup of the process, a continuous configuration is stable, ensuring product specifications over time (with a salt content lower than 0.03 M for both acid and base), especially when used in a treatment chain (as in the Water-Mining project system) or in an acid and base industrial production framework. Future research will focus on the economic evaluation of various process layouts to reduce production costs and make the EDBM technology market competitive.

## Conclusions

The present work provided an in-depth overview of the design, commissioning, and operational activities of the largest pilot-scale electrodialysis unit with bipolar membranes both in terms of membrane area and number of triplet for the production of hydrochloric acid and sodium hydroxide from synthetic brines. For the first time, three different process configurations for this technology were implemented and compared at the same process target. The pilot exhibited high stability in producing a NaOH target concentration of 1 M regardless of the operation mode adopted (closed-loop, feed and bleed, and fed-batch) and at current densities of 200 or 400 A m^–2^ for all configurations, as well as 300 and 500 A m^–2^ for closed-loop and feed and bleed modes. The results demonstrate that the operating conditions influence the behavior of the EDBM unit, resulting in different outcomes depending on the process configuration used. The EDBM pilot, when run in closed-loop mode (discontinuous mode) at 200 A m^–2^, produced the lowest SEC (among the three configurations) of 1.4 kWh kg^–1^ and the highest current efficiency of 80%. When the current density was increased, the feed and bleed configuration (continuous mode) was preferable. Indeed, low values of SEC (1.9–2.6 kWh kg^–1^), as well as high values of SP (0.82–1.3 ton year^–1^ m^–2^) and current efficiency (63–67%), were obtained. This study suggests that a continuous configuration should be preferred in an industrial context where acid and base production can be maximized using high current densities. Future research may focus on the use of real waste brines and on the economic evaluation of the various process configurations.

## Supporting Information Available

Schematic representation of an EDBM unit (Figure S1); summary of data reported in literature and comparison with those found in this work; water-mining project main features and role of the EDBM unit; properties and features of the employed IEMs; list of the pilot instruments and pumps; pH versus time trend for salt and ERS stream in the closed-loop configuration; results at 300 and 500 A m^–2^; and comparison among the three configurations (PDF)

## Abbreviations

AEM: anion-exchange membrane
BPM: bipolar membrane
CE: current efficiency
CEM: cation-exchange membrane
DSA: dimensionally stable anode
EDBM: electrodialysis with bipolar membrane
ERS: electrode rinse solution
HDPE: high-density polyethylene
MD: membrane distillation
MDC: membrane distillation crystallizer
MED: multieffective distillation
MLD: minimum liquid discharge
MMF: multimedia filtration
MSF: multistage flashing
PE: polyethylene
PEEK: polyether ether ketone
PET: poly(ethylene terephthalate)
PFD: process flow diagram
PK: aliphatic polyketone
PP: polypropylene
RED: reverse electrodialysis
RO: reverse osmosis
SEC: specific energy consumption
SP: specific production
SWRO: seawater reverse osmosis
ZLD: zero liquid discharge
**Symbols**
*C*_p,out_ (mol L^–1^): outlet concentration of reagent in feed and bleed mode
*C*_p,*t*_ (mol L^–1^): concentration of product at time *t*
*C*_r,0_ (mol L^–1^): concentration of reagent at time 0
*M*_p_ (g mol^–1^): molar weight
*F* (C mol^–1^): Faraday’s constant
*A*_m_ (m^2^): membrane area
*i* (A m^–2^): current density
*N*_tr_ (−): number of triplet
*Q* (L min^–1^): volumetric flow rate fed to the stack in the closed-loop and fed-batch
*C* (mol L^–1^): concentration
*Q*_bleed_ (L min^–1^): outlet volumetric flow rate in F&B mode
*Q*_r_ (L min^–1^): volumetric flow rate of reactant at time 0
*U* (V): voltage applied to the stack
*V*_p,0_ (m^3^): volume of product at time 0
*t* (s): time
*Q*_water_ (L min^–1^): makeup water volumetric flow rate in fed-batch mode
*V*_p,*t*_ (m^3^): volume of product at time *t*
Δ*t* (s): process time
τ_p_ (−): yield
*C*_p,0_ (mol L^–1^): concentration of product at time 0
*C*_p,in_ (mol L^–1^): inlet concentration of reagent in feed and bleed mode
*V*_r,0_ (m^3^): volume of reagent at time 0
*Q*_p_ (L min^–1^): volumetric flow rate of product at time *t*
