# Supplementary material for: Electrodialysis with Bipolar Membranes for the Sustainable Production of Chemicals from Seawater Brines at Pilot Plant Scale
Source: ACS Sustain Chem Eng. 2023 Feb 9;11(7):2989–3000. doi: 10.1021/acssuschemeng.2c06636 (PMC9945178; doi:10.1021/acssuschemeng.2c06636)
Supplement: Supplementary file 1 — sc2c06636_si_001.pdf [file sc2c06636_si_001.pdf]

SUPPORTING INFORMATION

Electrodialysis with bipolar Membranes for the sustainable production of chemicals from seawater brines at pilot plant scale

Calogero Cassaro †, Giovanni Virruso †, Andrea Culcasi †, Andrea Cipollina †, Alessandro Tamburini †*, Giorgio Micale †

† Dipartimento di ingegneria, Università degli studi di Palermo, Viale delle scienze Ed. 6, Palermo, 90128, Italia

*corresponding author: alessandro.tamburini@unipa.it

Number of pages - 9

Number of Tables - 4

Number of Figures - 7

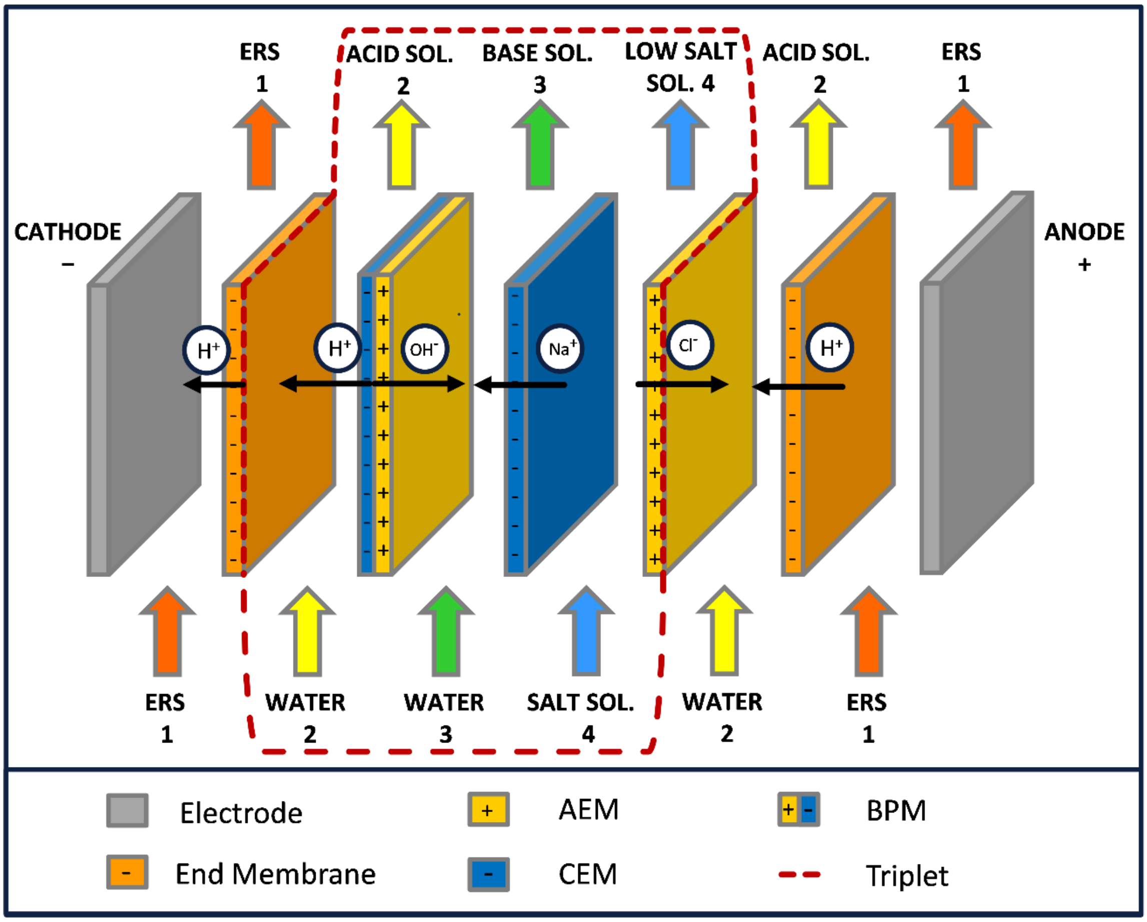

Figure S1. Schematic representation of an EDBM unit. The broken red line indicates the repetitive unit (i.e., the triplet) of the EDBM stack.

Table S1. Summary of operating conditions, concentrations and performance parameters for previously reported tests. ^{*a} , ^{*b} and ^{*s} refer to acid, base and salt, respectively.

**Process configuration**	**A_m m^2**	**i A m^{-2}**	**N_{tr}**	**Brine type**	**C mol l^{-1}**	**CE %**	**SEC kWh kg^{-1}**	**Ref**
Closed-loop	0.32	200-500	20	Synthetic (NaCl)	1^{*s} 0.90-0.95^{*a} 1.0-1.1^{*b}	61-71^{*a} 68-80^{*b}	1.7-3^{*a} 1.4-2.4^{*b}	This work
Feed & Bleed	0.32	200-500	20	Synthetic (NaCl)	1^{*s} 1.0-1.1^{*b}	60-66^{*b}	1.8-2.6^{*b}	This work
Fed-Batch	0.32	200-400	20	Synthetic (NaCl)	1^{*s} 0.86-0.95^{*a} 1.0-1.1^{*b}	53-48^{*a} 63-60^{*b}	2.4-2.9^{*a} 1.7-2.4^{*b}	This work
Closed-loop	0.0064	410-590	3	Synthetic (NaCl)	0.91-3.2^{*s} 0.88-2.2 ^{*a} 0.66-2.1^{*b}	55-88$^{*a\ b}$	1.8-3.6^{*a} 1.9-3.8^{*b}	[12]
Closed-loop	0.01	250-1000	2	Synthetic SWRO brine pre-treated	0.9^{*s} 0.6-0.8^{*a} 0.8-1^{*b}	45-70*a 55-80^{*b}	n.a	[38]
Feed & Bleed	0.0088	570	3	SWRO brine pre-treated	0.65^{*s} 1^{*a}	54^{*a}	7.6^{*a}	[39]
Two-stage-Closed-loop	0.0189	500	4	Synthetic (NaCl)	0.5^{*s} 1.2-1.6^{*a} 3.4^{*b}	29-37^{*a} 42-60^{*b}	2.4^{*a} 2.4-3.5^{*b}	[40]
Closed-loop	0.01	500-1000	1	Synthetic (NaCl)	1^{*s} 2.0-3.2^{*a} 2.9-3.6^{*b}	n.a	22-41^{*a}	[41]

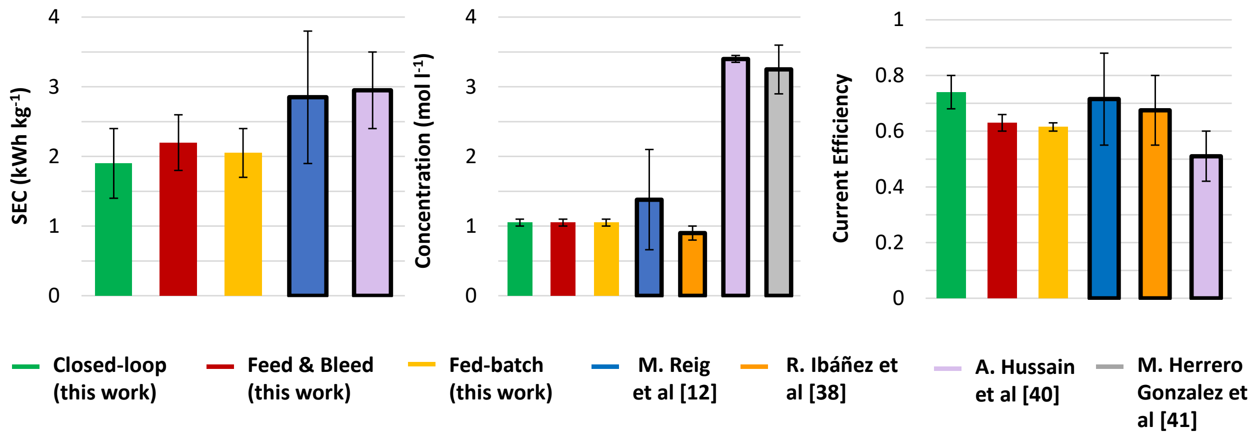

Figure S2. Comparison of the result found in this work with those already published in literature, in terms of specific energy consumption (SEC), concentration of the product and current efficiency. All the variables are referred to the base product.

<u>Role of EDBM in the Water-Mining project</u>

The Water-Mining project is an EU-funded multidisciplinary research project that creates water management solutions using a circular economy approach in the context of resource recovery systems. In this case, the EDBM unit is efficiently integrated into the system to produce valuable chemicals from waste saline solutions (Figure S4).

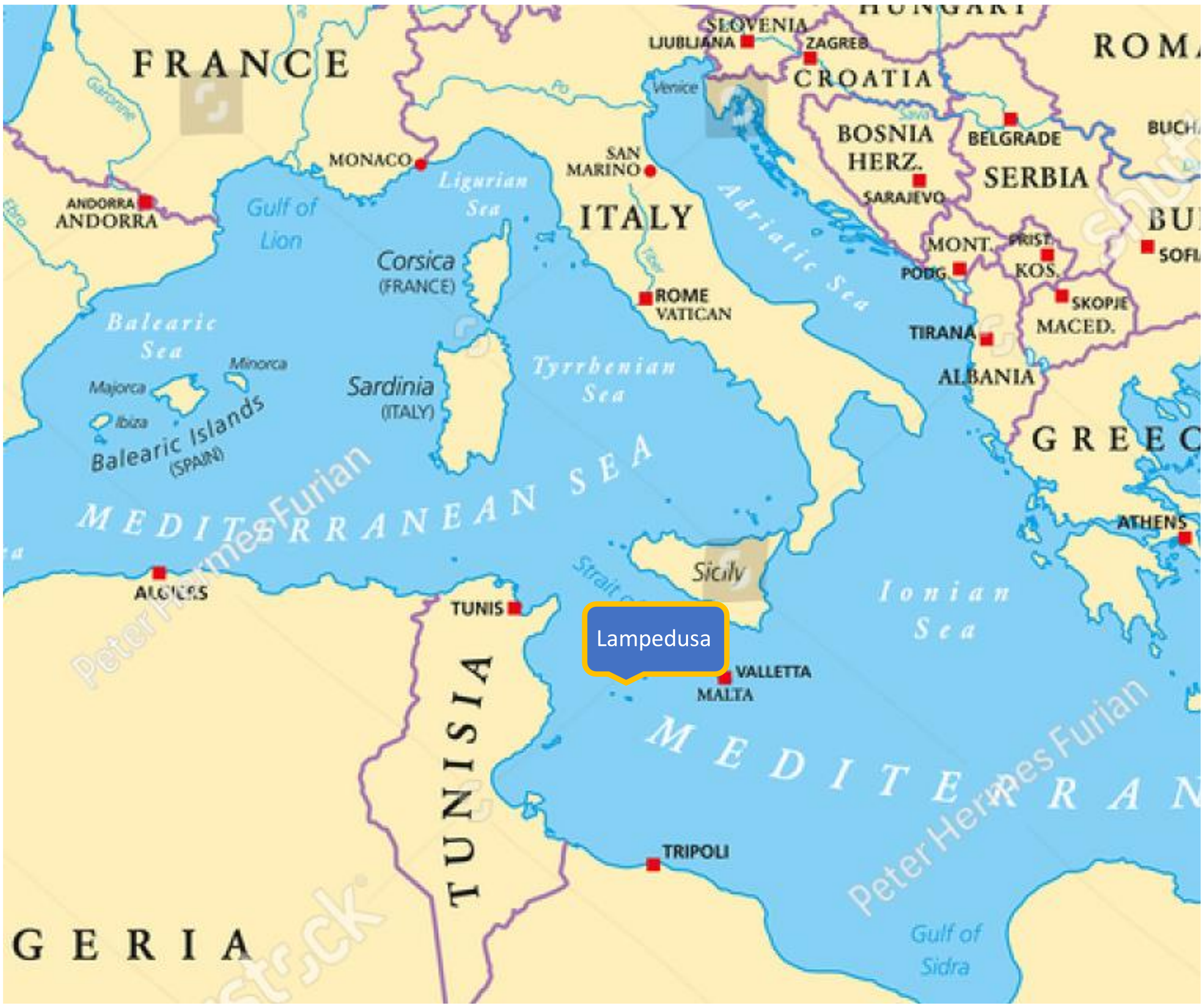

Figure S3. Geo-localization of the Water-Mining treatment chain.

The EDBM is fed with the Eutectic Freeze Crystallizer (EFC) brine effluent, which has a content of sodium chloride of about 1 M and trace of K^+, Mg^{2+}, Ca^{2+} and SO_4^{-2}. Electrodialysis with bipolar membrane (EDBM) promotes the circularity of resources by allowing the in situ production of chemical reagents useful for the other units of the treatment chain (Figure S4). Indeed, sodium hydroxide is used to generate $Mg(OH)_2$ and $Ca(OH)_2$ in the two stages of the MF-PFR pilot unit, whereas hydrochloric acid is used to perform membrane cleaning pre-treatments in all the membranes processes and to neutralize the MF-PFR effluent before sending it to the NF unit.

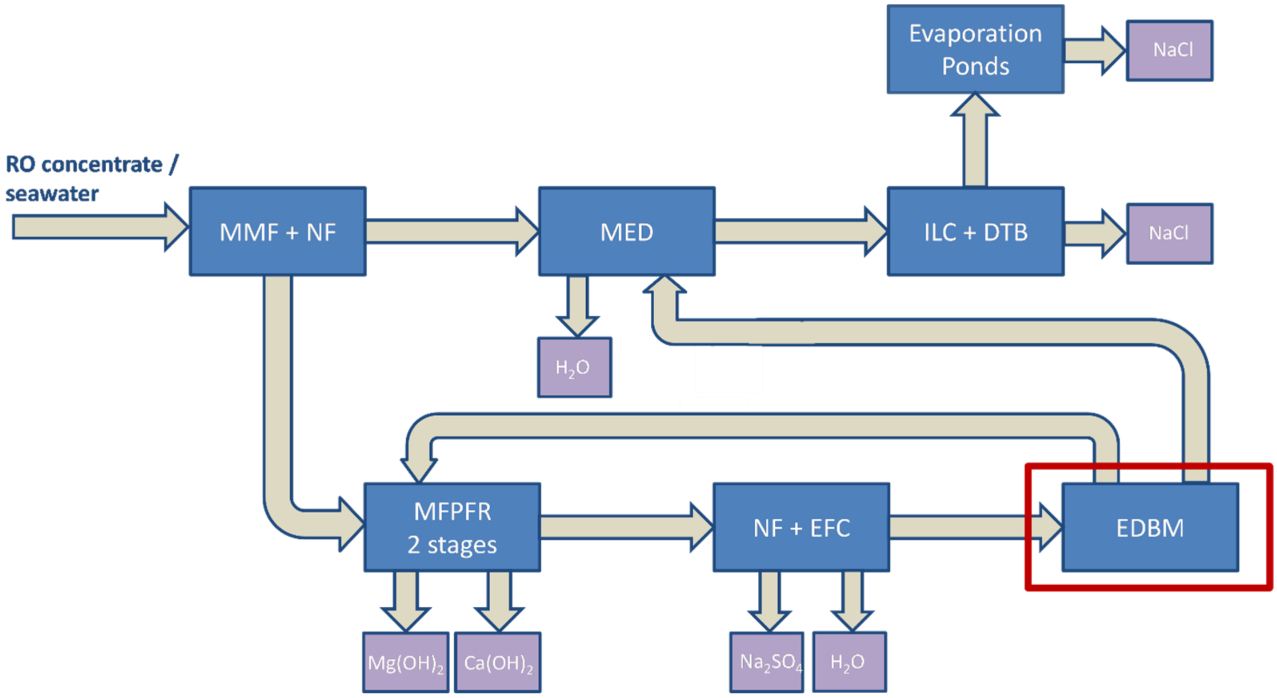

Figure S4. The Water-Mining treatment chain.

Table S2. List of typical properties of ion-exchange membranes used in EDBM units.

**Membrane**	**FAB-PK-130**	**FKL-PK-130**	**FBM**
Reinforcement	PEEK woven web	PEEK woven web	PEEK woven web
Resin type	Anion	Cation	Bipolar
Thickness	130 μm	130 μm	150 μm
Resistance (*)	<8.5 Ohm·cm^2	<10 Ohm·cm^2	n.a.
Selectivity (*)	>93%	>98%	n.a.
Swelling at 80°C	<2%	<4%	n.a.
E-Modulus	>1,000 MPa	>1,000 MPa	n.a.

(*) Measured in 0.5M NaCl solution

Table S3. List of the instruments and pumps installed in the EDBM pilot plant.

Element	Model	Range	Material	DN (mm)
Magnetic induction flowmeter	OPTIFLUX 4100C	0–30 l min^{-1}	PTFE	20
Conductivity meter	OPTISENS IND 1000	1–2000 mS cm^{-1}	PP	20
pH meter	SMARTPAT PH 8320	0–14	Glass AH	15
Pressure transducer	OPTIBAR P 1010 C	0–6 bar	AISI 316L	15
Turbine pump	TEOREMA PTM 2.5x6	0–3,500 rpm	PP	25

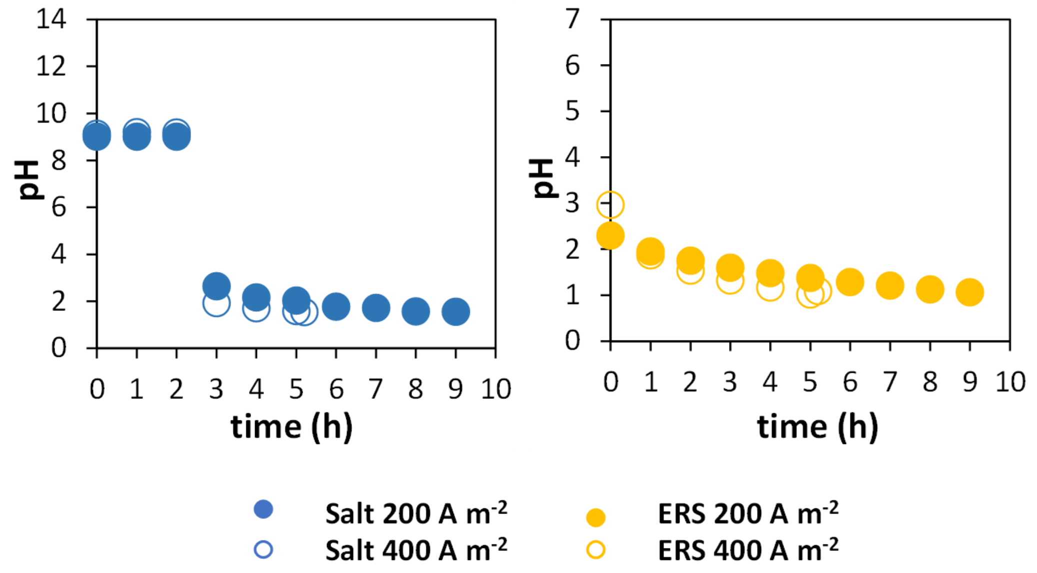

Figure S5. Dynamic trends of the pH inside the salt and ERS solutions for the tests in closed-loop at 200 and 400 A m^{-2}.

Table S4. Summary of the results obtained in feed & bleed configuration at 300 and 500 A m^{-2}.

i (A m^{-2})	Q_{bleed} (l min^{-1})	CE (%)	SEC (kWh kg^{-1})	SP (ton y^{-1}m^{-2})	C (mol l^{-1})
300	0.75	67.1	1.90	0.82	1.07
500	1.2	62.7	2.61	1.28	1.05

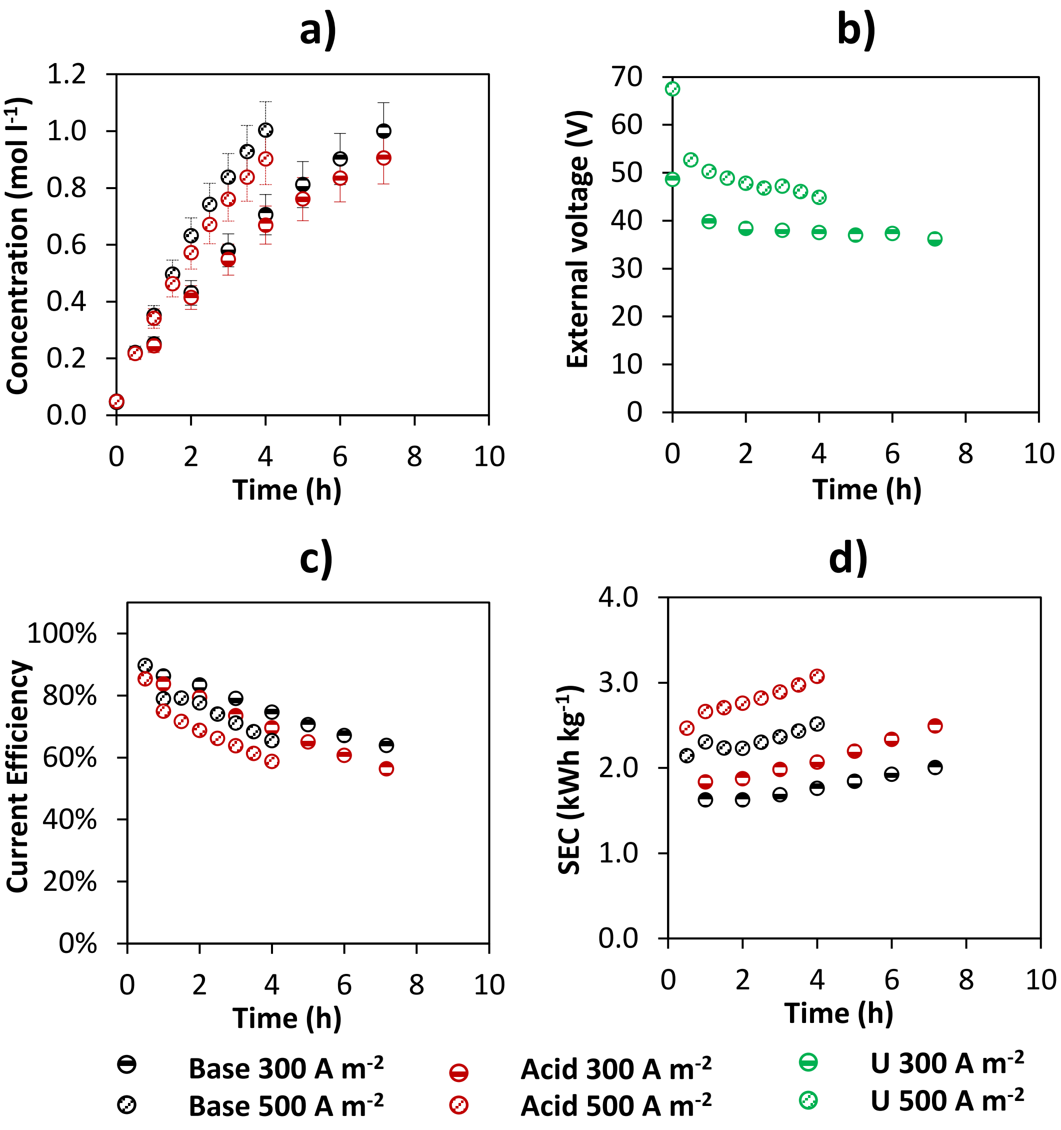

Figure S6. Time-dependent profiles of a) HCl and NaOH concentrations, b) external voltage, c) Current Efficiency and d) Specific Energy Consumption for acid and base for tests performed at 300 A m^{-2} and 500 A m^{-2}. Pilot operation mode: closed-loop (batch).

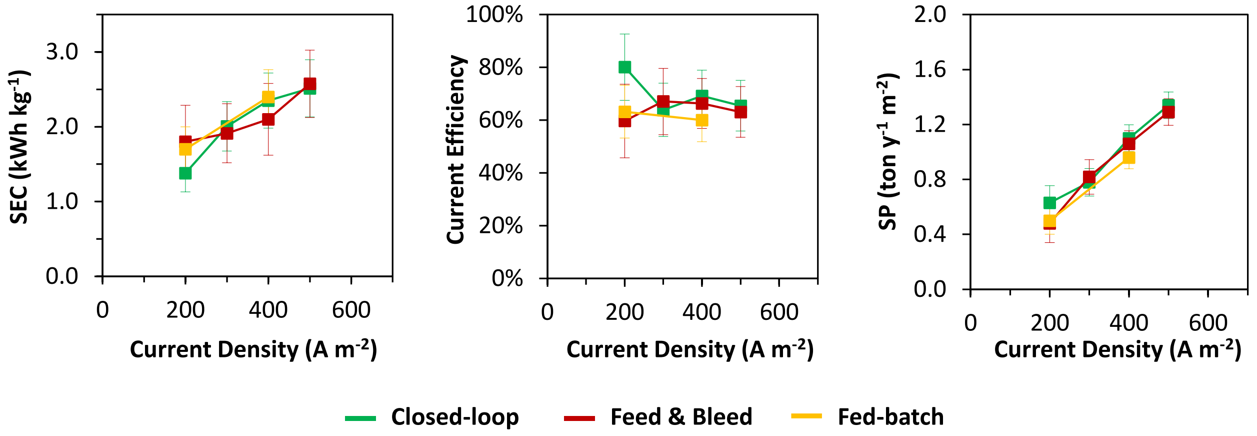

Figure S7. Trends of the main performance indicators for the three configurations at all investigated current density.
